# Risk factors for colonization by carbapenemase-producing bacteria in Spanish long-term care facilities: a multicentre point-prevalence study

**DOI:** 10.1186/s13756-022-01200-0

**Published:** 2022-12-20

**Authors:** Manuel Callejón Fernández, Ana Madueño Alonso, Rossana Abreu Rodríguez, Armando Aguirre-Jaime, María Beatriz Castro Hernández, María José Ramos-Real, Yanet Pedroso-Fernández, María Lecuona Fernández

**Affiliations:** 1 Microbiology and Infection Control Service, Hospital Universitario de Canarias, La Laguna, Tenerife, Spain; 2 Department of Preventive Medicine and Public Health, University of La Laguna, Tenerife, Spain; 3 Institute of Care Research, Nurses Association of Santa Cruz de Tenerife, Tenerife, Spain

**Keywords:** Carbapenemase, Colonization, Long term care facilities, Multidrug-resistant organism, Risk factors, Prevalence

## Abstract

**Background:**

The emergence of carbapenemase-producing bacteria (CPB) has become a major public health concern. Long-term care facilities (LTCF) are potential reservoirs for multidrug-resistant micro-organisms (MDRO). However, data on CPB is limited. The study aims to determine the prevalence of MDRO and risk factors for CPB colonization among residents of LTCFs.

**Methods:**

A point-prevalence study was conducted at 14 LTCFs in Tenerife (Spain) between October 2020 and May 2021. Nasal and rectal swabs were cultured for methicillin-resistant *Staphylococcus aureus* (MRSA), vancomycin-resistant enterococci (VRE), carbapenemase-producing *Enterobacterales,* MDR *Acinetobacter baumannii* (MDR-Ab) and MDR *Pseudomonas aeruginosa*. Antimicrobial susceptibility testing and molecular detection of resistance genes were performed. Risk factors for colonization by carbapenemase-producing bacteria (CPB) were determined by univariate and multivariate analysis.

**Results:**

A total of 760 LTCF residents were recruited. The prevalence of colonization by CPB was 9.3% (n = 71) with the following distribution: 35 (49.3%) *K. pneumoniae*, 26 (36.6%) MDR-Ab, 17 (23.9%) *E. coli*, and 1 (1.4%) *C. koseri*. In addition, the prevalence of colonization by MRSA was 28.1% (n = 215) and only one case of VRE was isolated. Multivariate analysis identified male sex (odds ratio [OR], 1.86; 95% confidence interval [CI], 1.86–3.11; *P* = 0.01), having a high health requirement (OR, 6.32; 95% CI, 1.91–20.92; *P* = 0.003) and previous hospitalization (OR, 3.60; 95% CI, 1.59–8.15 *P* = 0.002) as independent risk factors for CPB rectal carriage.

**Conclusions:**

LTCFs are an important reservoir for MDRO, including CPB. We have identified some predictors of colonization by CPB, which enable a more targeted management of high-risk residents. Antimicrobial stewardship programmes and infection control preventive measures are needed to stop acquisition and transmission of MDRO.

## Background

The emergence of multidrug-resistant organisms (MDROs) is a global public health problem [1]. Initially, many of these MDROs appeared to cause hospital-acquired infections [2], but more recently they have spread into different healthcare settings, including long-term care facilities (LTCFs) [3, 4], and also into the community [5]. LTCFs are recognized as an important reservoir of methicillin-resistant *Staphylococcus aureus* (MRSA) and extended-spectrum β-lactamase *Enterobacterales* (ESBL) [6]. Recently there has been growing interest in knowing the prevalence of colonization by other MDROs such as carbapenemase-producing *Enterobacterales* (CPE), vancomycin-resistant *Enterococcus* spp (VRE), MDR *Acinetobacter baumannii* (MDR-Ab) and MDR *Pseudomonas aeruginosa* (MDR-Pa) [7–15]. Specifically, the increasing prevalence of infections by MDR gram negative bacteria (MDR- GNB) have become a real threat in recent years. Moreover, there is a risk of LTCFs becoming a reservoir for these pathogens [16, 17].

LTCFs provide residential healthcare for people with significant disabilities, chronic illness and elderly individuals who cannot care for themselves. These institutions are also the last medical resource for patients who have survived acute illnesses in hospitals. The increase in life expectancy and, therefore, ageing of the population has meant that LTCFs have become essential in the healthcare system. However, there is evidence that a stay in a LTCF is a risk factor for the carriage of MDROs [18, 19] and this can be for several reasons: high pressure antibiotics, permanent living in a confined environment, the difficulty of diagnosing infections that present atypically and common cognitive impairment. In addition, it has been demonstrated that continuous bidirectional movement of patients between these institutions and acute care hospitals facilitates the spread and maintenance of MDRO bacteria [20, 21]. For these reasons, identifying the patients who carry MDROs and preventing the hospital from nosocomial spreading is challenging.

Antimicrobial stewardship programmes (ASP) have been widely implemented in hospitals [22], in addition to monitoring and prevention programmes with the aim of reducing the incidence of these infections. LTCFs could also benefit from these programmes. Knowledge of the epidemiology of MDROs at local level is key to implementing a successful antimicrobial stewardship intervention. However, the prevalence of MDR GNB faecal carriage in LTCFs remains unknown in most geographical areas [12].

The aim of this study is to determine the prevalence of MDROs and risk factors for colonization by carbapenemase-producing bacteria (CPB) among LTCF residents in North Tenerife (Spain). In addition, the MDROs resistance mechanism was characterized in molecular terms.

## Methods

### Study design

A multicentre point-prevalence study (October 2020–May 2021) was conducted at 14 LTCFs distributed throughout North Tenerife (Spain). In each LTCF, rectal and nasal swab were collected from all residents. The residents’ sociodemographic and clinical data were evaluated by means of a questionnaire. None of the LTCFs taking part had a specific action protocol to monitor and prevent MDRO transmission. A resident colonized by CPB was defined as a case, and a control was defined as those who were not colonized by CPB.

### Microbiological methods

All samples were analyzed at the Microbiology Service in Hospital Universitario de Canarias, which is the reference hospital in the northern area of Tenerife. Rectal swabs were cultured directly on selective chromogenic media ChromID® CARBA SMART and ChromID® VRE (bioMèrieux, Marcy l´Etoile, France) and McConkey (bioMèrieux). Nasal swabs were cultured on ChromID® MRSA SMART (bioMèrieux) and inoculated into Brain–Heart Infusion Broth (bioMèrieux). They were reseeded in MRSA after 24 h of incubation in broth.

Identification and antimicrobial susceptibility testing of the suspicious colonies were performed with the Vitek-II® system (bioMèrieux) and reduced susceptibility/resistance to imipenem or vancomycin was confirmed by Etest (bioMèrieux). Carbapenemase production was phenotypically tested by the agar tablet/disc diffusion method (KPC/MLB and OXA-48 ConfirmKit; ROSCO Diagnostica, Taastrup, Denmark). Colistin resistance was tested by disk diffusion test and confirmed by broth microdilution (UMIC, Biocentric, France). All results were analyzed and interpreted according to the European Committee on Antimicrobial Susceptibility Testing guidelines [23]. Colonies suspected of MRSA were confirmed by the PBP2A SA culture colony Test (AlereTM Scarborough, Maine, USA).

Genes for resistance to carbapenems (NDM, VIM, KPC, OXA-48, IMP) for *Enterobacterales* and *P. aeruginosa*; and to vancomycin for enterococcus (vanA, vanB) were genotypically characterized by multiplex polymerase chain reaction (PCR) AllplexTM Entero-DR Assay (Seegene, Korea). Carbapenemase resistance in *A. baumannii* (OXA-51, NDM, OXA-23, OXA-40, OXA-58) and the detection of methicillin resistance genes for *S. aureus* (mecA, mecC) was characterized by isothermal amplification Eazyplex® with Superbug Acineto and MRSA reagents, respectively (AmplexDiagnostics, Germany).

### Statistical analysis

The sample collected from 71 cases and 689 controls offers the study a power of 90% in detecting a difference between cases and controls of relative frequencies for nominal variables of at least 20%; or 3 years for the age or days for the stay in ranges of 0–5 in bilateral tests of hypothesis at a level of statistical significance *P* ≤ 0.05.

The characteristics of the sample as a whole are reported by summarizing its nominal variables with the frequency (relative frequency) of its component categories, and those of numerical scale with mean (P_5_–P_95_) given its distance from a normal probability distribution verified with the Kolmogorov–Smirnov test.

Nominal variable cases and controls are compared with Pearson’s χ^2^ test or Fisher's Exact Test. Numerical scale comparisons were made with the Mann–Whitney U test. Those variables that in these comparisons attained a significance of at least 5% in their difference will enter as potentially predictive factors of a combined colonization by CPB as an effect, first in univariate logistic regression models and then in a regression model backward stepwise multivariate binary logistics using the Wald criterion to estimate odds ratios for independent predictors of colonization.

All hypothesis contrast tests are two-sided at a level of statistical significance *P* ≤ 0.05 and the calculations involved in these operations are executed with the help of the statistical package for statistical data processing SPSS 25.0™ from IBM Co.® (IBM –SPSS Inc, Armonk, NY, USA).

## Results

Among 14 LTCFs (10 publics and 4 private), 764 residents were selected to participate in the study. However, we failed to obtain rectal samples from four residents, resulting in a total 760 residents included in the study (Table 1). A total of 71 (9.3%) and 689 (90.7%) residents were classified as cases and controls, respectively. Cases were colonized by CPB in the following proportions: 35 (49.3%) *K. pneumoniae*, 26 (36.6%) MDR-Ab, 17 (23.9%) *E.coli* and 1 (1.4%) *C. koseri*. In addition, 26 (36.6%) were also colonized by MRSA and two (2.8%) by MDR-Pa. Of the controls, 188 (27.3%), 12 (1.7%) and 1 (0.1%) were colonized by MRSA, MDR-Pa and VRE, respectively. The results obtained in terms of characterization of resistance mechanism are shown in Table 2.

**Table 1:** Distribution of residents by the different LTCFs included in the study

LTCF	No. beds	No. recruited residents	No. residents colonized by CPB
LTCF-A	99	43 (43.4%)	2 (4.7%)
LTCF-B	99	71 (71.7%)	10 (14.1%)
LTCF-C	193	70 (36.3%)	12 (17.1%)
LTCF-D	102	70 (68.6%)	1 (1.4%)
LTCF-E	32	32 (100%)	0 (0%)
LTCF-F	86	78 (90.7%)	12 (15.4%)
LTCF-G	75	74 (98.7%)	4 (5.4%)
LTCF-H	20	20 (100%)	0 (0%)
LTCF-I	60	35 (58.3%)	0 (0%)
LTCF-J	130	127 (97.7%)	7 (5.5%)
LTCF-K	37	10 (27%)	1 (1%)
LTCF-L	600	71 (11.8%)	15 (21.1%)
LTCF-M	60	36 (60%)	6 (16.6%)
LTCF-N	48	23 (47.9%)	1 (4.4%)

**Table 2:** Mechanism of resistance of MDROS isolates (cases and controls)

	*MRSA* N = 215	*VRE* N = 1	A.*baumannii* B.N = 26	*K. pneumoniae*^a^ N = 35	*E. coli* N = 17	*C. koseri* N = 1	*P. aeruginosa* N = 14
mecA	215 (100%)						
vanA		1 (100%)					
OXA-51			26 (100%)				
OXA-58			26 (100%)				
OXA-48, CTX-M				22 (62.9%)	6 (35.3%)		
OXA-48				8 (22.9%)	11 (64.7%)	1 (100%)	
KPC				3 (8.6%)			
KPC, CTX-M				1 (2.9%)			
VIM							1 (7.1%)

^a^The resistance mechanism study could not be performed on a strain of *K. pneumoniae* by molecular biology

The clinical and epidemiological characteristics of the two groups are shown in Table 3. Cases were significantly more likely than controls to be male (*P* = 0.025), have active infection (*P* = 0.025), urinary incontinence (*P* = 0.044), faecal incontinence (*P* = 0.014), previous antibiotic use (*P* = 0.040), high/medium health requirement (*P* = 0.005/0.05), prior hospital admission within the last 3 months (*P* = 0.002) and previous MDRO (*P* = 0.013).

**Table 3:** Comparison of potential predictive factors for CPB colonization between resident cases and controls

Variable	Cases (n = 71)	Controls (n = 689)	*P* value
Age (years)	81 (53–96)	83 (58–95)	0.125
Male (sex)	32 (45.1)	219 (31.8)	0.023
Single room	6 (8.5)	87 (12.6)	0.320
Length of stay at LTCF (days)	1122 (16–6639)	1163 (69- 5170)	0.178
*Intrinsic risk factors*			
Diabetes mellitus	23 (32.4)	236 (34.3)	0.795
Dermatitis	25 (35.2)	195 (28.3)	0.201
Peripheral vascular disease	20 (28.2)	156 (22.6)	0.272
Chronic kidney disease	3 (4.2)	55 (7.9)	0.264
Chronic obstructive pulmonary disease	11 (15.5)	85 (12.3)	0.426
Active infection	19 (26.8)	112 (16.3)	0.023
Urinary incontinence	59 (83.1)	501 (72.8)	0.041
Faecal incontinence	53 (74.7)	415 (60.2)	0.013
*Extrinsic risk factors*			
Dialysis	0	3 (0.4)	0.987
Central venous catheter	0	7 (1)	0.968
Urinary catheter	4 (5.6)	17 (2.5)	0.121
Feeding tubes	5 (7)	26 (3.8)	0.196
Previous antibiotic use^a^	37 (52.1)	275 (39.9)	0.039
Health requirement^b^			
High	45 (63.4)	322 (46.7)	
Medium	21 (29.6)	242 (35.1)	0.004
Low	3 (4.2)	118 (17.1)	
Prior hospital admission^a^	10 (14.1)	32 (4.6)	0.003
Length of hospital stay (days)	1 (1–62)	2 (1–38)	0.257
Previous MDRO colonization^c^	26 (36.6)	158 (22.9)	0.012

Values are shown as mean (P5–P95) or n (%)

^a^During the previous 3 months prior to study recruitment

^b^According to local guides [24]

^c^The resident has a history of MDRO carriage/infection (past year). MDROs included methicillin-resistant Staphylococcus aureus, imipenem-resistant Acinetobacter baumannii, vancomycin-resistant Enterococcus, multidrug-resistant Pseudomonas aeruginosa and carbapenemase-producing Enterobacterales

On multivariate analysis (Table 4), the only variables retained as independent risk factors for colonization by CPB were male sex (OR, 1.86; 95% CI, 1.86–3.11; *P* = 0.01), high/medium health requirement (OR, 6.32; 95% CI, 1.91–20.92; *P* = 0.003/OR, 3.78; 95% CI,1.09–13.04; *P* = 0.036) and previous hospitalization (OR, 3.60; 95% CI, 1.59–8.15; *P* = 0.002).

**Table 4:** Univariate and multivariate analyses for risk factors associated with carriage of CPB

Variable	OR (95% CI) univariate	*P* value	OR (95%CI) multivariate	*P* value
Male (sex)	1.76 (1.07–2.89)	0.025	1.86 (1.11–3.11)	0.018
Active infection	1.91 (1.08–3.35)	0.025		
Urinary incontinence	1.98 (1.02–3.85)	0.044		
Faecal incontinence	2.04 (1.15–3.59)	0.014		
*Health requirement*				
Low	Reference		Reference	
Medium	3.41 (1–11.67)	0.050	3.78 (1.09–13.04)	0.036
High	5.50 (1.68–18)	0.005	6.32 (1.91–20.92)	0.003
Previous antibiotic use	1.68 (1.02–2.74)	0.040		
Prior hospital admission	3.41 (1.60–7.27)	0.002	3.60 (1.59–8.15)	0.002
Previous MDRO colonization	1.92 (1.15–3.21)	0.013		

*CI* confidence interval, *OR* odds ratio

## Discussion

This study was conducted due to the increasing prevalence of infections by CPBs in acute care hospitals in our geographical area. We sought to know whether these LTCFs also constitute a reservoir of CPB, in addition to identifying risk factors for colonization by CPB.

In this multicentre point-prevalence study, a remarkably high rate of colonization by CPB was observed among LTCF residents in North Tenerife. In the literature, there are few recent studies about CPB in Europe showing a high geographical variation [8, 12]. Most studies reported low CPB prevalence rates (0.06–1.7%) among residents of LTCFs [11, 13, 24–26]. However, some studies in Israel (12%), Spain (4.1%) and Italy (28.4%) determined a high prevalence [4, 17, 27]. Our findings are in line with other studies in Spain, which confirm that LTCFs are turning into reservoirs of CPB [16].

Our multivariate analysis identified male sex, a high or medium health requirement and previous hospitalization as important risk factors for CPB rectal colonization. Several studies have identified male sex as a risk factor for MDRO colonization [21, 28]. However, the reason why male sex is a risk factor remains unknown. Rodríguez-Villadores et al. [8] explain that this may be due to a higher frequency of risk factors among male residents, who have more comorbidities compared to female residents.

Establishing a level of dependence for the probability of MDRO colonization is difficult, mainly due to the variability of scores used across the studies. Some commonly used methods are Katz, Barthel, Karnofsky or the French index. In our study, all LTCFs have a bed distribution according to health requirements (high, medium or low) and, therefore, residents were classified according to the criteria of the centres themselves. Despite the lack of a dependence threshold related to MDRO colonization, there appears to be compelling evidence to indicate that an increased level of dependence is associated with an increased risk of being colonized by MDROs [8].

Several previous studies have identified prior hospital admission as a risk factor for MDRO colonization [12, 29]. Depending on the study, they establish the limit at three, six or twelve previous months. As in our study, hospital admission in the previous 3 months was also reported to be a risk factor for MDRO colonization [30]. It remains unknown how long after hospital admission this risk increases. Therefore, previous hospitalization should be considered a risk factor for MDRO colonization among LTCF residents. However, to what extent this risk could be increased by days of hospitalization remains unknown [8].

Our univariate analysis identified active infection, urinary and faecal incontinence, previous antibiotic use and previous MDRO colonization as factors associated with colonization by CPB. However, multivariate analysis did not reveal these to be independent risk factors. These are traditional factors associated with MDRO colonization in the literature and previous use of antibiotics is the main associated factor [6, 31–33].

Since the aim of the study was to assess colonization factors exclusively by CPB and control residents by MDR-Pa and VRE were scarce, we decided to include them in the statistical analysis. Nevertheless, we performed this analysis without including theses control residents and obtained the same significant risk factors.

This study has several limitations. First, as the study was performed during the COVID-19 pandemic, we had enormous difficulty accessing the centres for sample collection and filling out the questionnaires, delaying the study deadlines. Second, the cross-sectional survey design did not enable us to investigate the dynamic of MDRO colonization (acquisition, persistence and clearance of carriage). Third, disk diffusion by colistin did not allow the detection of resistance to this antibiotic since there are not breakpoints for EUCAST and CLSI, requiring confirmation by another technique.

## Conclusions

Our study documents a high prevalence of colonization by MDROs, including CPB, among LTCF residents in North Tenerife. This emphasizes the role of these centres as reservoirs for MDROs. Male sex, a high health requirement and prior hospital admission were all identified as independent risk factors for CPB rectal colonization. These results strengthen the importance of establishing a standardized protocol to manage colonized patients between acute hospital centres and LTCFs. In addition, antimicrobial stewardship programmes and infection control preventive measures accounting for related risk factors in LTCFs are required to stop the acquisition and transmission of MDROs in healthcare facilities.

## Data Availability

The datasets generated and analyzed during the current study are not publicly available due to the requirement to protect patient confidentiality.

## Abbreviations

ASP: Antimicrobial stewardship programmes
CPB: Carbapenemase-producing-bacteria
CPE: Carbapenemase-producing *Enterobacterales*
ESBL: Extended-spectrum β-lactamase *Enterobacterales*
LTCF: Long-term care facilities
MDR: Multidrug-resistant
MDR-Ab: Multidrug-resistant *Acinetobacter* *baumannii*
MDR-GNB: Multidrug-resistant gram negative bacteria
MDRO: Multidrug-resistant micro-organisms
MDR-Pa: Multidrug-resistant *Pseudomonas aeruginosa*
MRSA: Methicillin-resistant *Staphylococcus aureus*
OR: Odds ratio
VRE: Vancomycin-resistant enterococci
